# Accuracy of Using Mid-Upper Arm Circumference to Detect Wasting Among Children Aged 6–59 Months in Nepal

**DOI:** 10.9745/GHSP-D-20-00450

**Published:** 2021-12-31

**Authors:** Krishna Prasad Lamsal, Kedar Raj Parajuli, Bhim Kumari Pun, Ramesh Prasad Adhikari, Manoj Bashyal, Baburaja Dangol, Kenda Cunningham

**Affiliations:** a Nutritionist, Kathmandu, Nepal.; b Nutrition Section, Ministry of Health and Population, Government of Nepal, Kathmandu, Nepal.; c Helen Keller International, Kathmandu, Nepal.; d Nepali Technical Assistance Group (NTAG), Kathmandu, Nepal.; e NewEra, Kathmandu, Nepal.; f London School of Hygiene and Tropical Medicine. London, United Kingdom.

## Abstract

**Background:**

In countries with a high prevalence of undernutrition, timely, accurate screening at the community level is essential to identify children with wasting. The World Health Organization recommends using either weight-for-height z-scores (WHZ) and mid-upper arm circumference (MUAC) or both measures and signs of edema to be used to identify children with severe acute malnutrition for treatment. We compared WHZ and MUAC cutoffs to identify wasting among children aged 6–59 months in Nepal, using WHZ as the reference standard.

**Methods:**

We used cross-sectional anthropometric data for 3,169 children aged 6–59 months from a 2017 cross-sectional dataset, representative of 42 of Nepal's 77 districts. We used descriptive statistics, receiver operating characteristic (ROC) curves, and kappa statistics to compare the use of MUAC and WHZ to identify wasting. The Youden index was calculated to determine the optimum MUAC cutoffs.

**Results:**

The prevalence of wasting was 3.1% and 10.5% using MUAC and WHZ, respectively. We found 13.6% sensitivity for severe acute malnutrition (SAM) (MUAC <115 mm) and 21.0% sensitivity for moderate acute malnutrition (MAM) (MUAC ≥115 to <125 mm), with specificity of 99.7% and 91.2%, respectively. The sensitivity of MUAC for children aged 6–23 months was higher than for children aged 24–59 months. The total area of the ROC curve was 0.53 for the MUAC cutoff for SAM and 0.56 for MAM. The optimum MUAC cutoffs for SAM and MAM were 125 mm and 132 mm, respectively.

**Conclusions:**

Although MUAC can be used as a rapid screening tool to detect wasting in children aged 6–59 months, using the recommended MUAC cutoffs captures only a small proportion of the total number of wasted children. The poor sensitivity and specificity of MUAC compared to WHZ suggests a need to refine admission and discharge criteria for acute malnutrition management programs to ensure that wasting among infants and children in Nepal is consistently and accurately diagnosed and treated.

## Abstract (precis)

When comparing the sensitivity and specificity of mid-upper arm circumference (MUAC) versus weight-for-height z-scores (WHZ) to identify wasting in children aged 6–59 months in Nepal, our findings suggest that only using MUAC compared to WHZ to screen may exclude a large number of children who could be at risk of severe or moderate acute malnutrition.

## Teaser

**Key Finding:**

- Mid-upper arm circumference (MUAC) had a lower sensitivity compared to weight-for-height z-score (WHZ), indicating it can detect only a small proportion of the total number of children aged 6–59 months who are wasted.

**Key Implication:**

- Using sensitivity and specificity criteria, the poor performance of MUAC compared to WHZ to identify cases of severe and moderate wasting in infants and children aged 6–59 months suggests a need to further refine admission criteria, including the choice of indicators and cutoffs.

## INTRODUCTION

Undernutrition has devastating individual and public health consequences: it weakens individuals' immune systems, worsens illnesses for individuals, and is linked to poor economic growth and poverty. Furthermore, undernourished children who survive have diminished learning capacity and lower productivity in adulthood.^1^ Acute malnutrition, especially severe acute malnutrition (SAM), is an unstable condition resulting from a relatively short duration of nutritional deficit that is often complicated by concurrent infective illness. A severely wasted child has a more than 11-fold increased risk of death compared to a non-wasted child.^2^^–^^4^

In countries with a high prevalence of undernutrition, timely, accurate screening at the community level is essential to identify children with acute malnutrition. More than 500,000 deaths annually could be prevented globally by timely and proper treatment of acute malnutrition.^5^ Children aged 6–59 months who have a mid-upper arm circumference (MUAC) <115 mm, a weight-for-height z-score (WHZ) <–3 standard deviation (SD) (based on the World Health Organization [WHO] Child Growth Standards), and/or nutritional edema are considered to have SAM. Likewise, children aged 6–59 months having WHZ <−2 to ≥−3 SD and/or MUAC ≥115 mm to <125 mm are classified as having moderate acute malnutrition (MAM).^4^ MUAC is a widely used tool, especially in resource-limited countries to identify wasting and is closely associated with mortality risk. WHO recommends using either or both WHZ or MUAC and the presence of nutritional edema to assess the prevalence of acute malnutrition and for admission and graduation criteria for treatment programs.

The prevalence of child undernutrition in Nepal is among the highest in the world. Among children aged under 5 years, 10% are wasted (low weight-for-height) including 2% severely wasted; this alarmingly high level has persisted for at least 2 decades.^6^^,^^7^ To reduce the prevalence of wasting, the Government of Nepal, in collaboration with donors and nongovernmental partners, has been implementing community and health facility-based programs. Since 2009, when the community-based management of acute malnutrition (CMAM) program was piloted, more than 40,000 female community health volunteers have used only MUAC to find wasted children in their communities. In contrast, health facility workers in Nepal use both WHZ and MUAC as well as nutritional edema as criteria to admit children to outpatient therapeutic centers, stabilization centers, and nutrition rehabilitation homes. However, only MUAC is used as the measure for discharge in SAM management programs and as the admission and discharge measure for MAM management programs.^7^^,^^8^

Researchers have raised concern about the effectiveness of using MUAC as a wasting screening tool because of its low sensitivity compared to WHZ.^9^^–^^11^ As per Fernandez et al.^11^ using MUAC <115 mm identified only 10 of 165 children with WHZ <−3. Thus, using MUAC <115 mm during community-level screening would leave more than 90% of children with a WHZ <−3 without therapeutic treatment. In other words, only using MUAC to screen for malnutrition globally would result in 14.4 of the 16 million children in 2016 with SAM (defined as WHZ <−3) not identified or treated. Therefore, understanding how MUAC performs, versus WHZ, in the detection of wasting in different populations is important. Prior studies on this have been mostly hospital-based rather than community-based and most were conducted in Africa. To date, no studies have been conducted in Nepal and very few studies done in South Asia to assess the accuracy of MUAC versus WHZ for detection of wasting. Several studies have also raised questions on the appropriateness of using the same cutoff for all children aged 6–59 months, given some findings of age and gender variation.^12^^–^^15^ Because WHZ and MUAC are the major anthropometric tools used to identify wasting in children, comparing the accuracy of both tools to identify wasting provides a better sense of the accuracy of measurements. Few studies have been done to compare the diagnostic accuracy of MUAC by assuming WHZ as a gold standard.^10^^–^^12^^,^^15^ Using WHZ as the reference standard, we aimed to identify the magnitude of discrepancies in the identification of wasted children in Nepal by MUAC versus WHZ, whether these discrepancies vary by child age or gender, and ideal MUAC cutoffs for more thorough detection of children with SAM and MAM.

Only using MUAC to screen for malnutrition globally would result in 14.4 of the 16 million children in 2016 with SAM not identified or treated.

## METHODS

This study uses a cross-sectional dataset collected by a local survey firm in July–September 2017 across 16 of Nepal's 77 districts, including a total sample of 3,169 children aged 6–59 months. Structured questionnaires were used to interview the young child's mother and a household head (with preference given to a male decision maker, when available) to obtain socioeconomic and demographic information, as well as to measure knowledge and practices related to nutrition, reproductive health and family planning, agriculture and food security, empowerment, and exposure to health and nutrition-related interventions.

Anthropometry, including length/height, weight, and MUAC of each child, was measured by a team of 20 trained and standardized data collectors. The length of children aged 6–23 months and height of children aged 24–59 months were measured using the vertical stadiometer, following WHO guidelines.^16^ Weight was measured with an electronic digital weighing scale (Seca scale) and read to the nearest 0.1 kg with minimum light clothing. Calibration of the Seca scales was done before weighing each child. For MUAC measurement, we used nonstretchable and flexible MUAC tapes procured from United Nations Children's Fund. The measurement was taken at the midpoint of the acromion of the scapula and the olecranon of the ulna of a child. The height/length and MUAC of each child were measured twice and the measurements averaged to get the final raw length/height, weight, and MUAC measurements.

These anthropometric data were then transformed into WHZ z-scores. The prevalence of wasting was calculated by using both WHZ and MUAC. To classify children as SAM and MAM using WHZ, we used cutoffs of <-3 SD WHZ and ≥-3 to <-2 SD WHZ, respectively. To classify children as SAM and MAM using MUAC, we used cutoffs of <115 mm and ≥115 mm to <125 mm, respectively.^4^^,^^7^ We assumed WHZ as the reference measure and tested the sensitivity of MUAC cutoffs versus WHZ. The sensitivity and false-positive rates (1-specificity) of MUAC were determined using WHO classification for SAM and MAM. To assess the performance of MUAC cutoffs compared to the standard recommended by WHO to define SAM and MAM, receiver operating characteristic (ROC) curves were constructed and categorized as: 50%–60% very poor, 61%–70% poor, 71%–80% fair, 81%–90% good, and 91%–100% with excellent sensitivity. The ROC curve is the plot of sensitivity versus false-positive rate of MUAC cutoffs. The area under the curve (AUC) is the area between the curve and the segment (0,0) and (1,1), which corresponds to a random classifier. A larger AUC indicates a more accurate diagnosis of acute malnutrition, defined by WHZ cutoffs.^5^^,^^10^^,^^17^^,^^18^ Kappa statistic was used to analyze inter-rater agreement between MUAC and WHZ by assuming: <0% as none, 0%–20% as poor, 21%–40% as slight, 41%–60% as fair, 61%–80% as good, 81%–92% as very good, and 93% or more as excellent agreement.^19^ To identify the MUAC cutoff with the highest sensitivity and specificity, the Youden index, which is the difference between the true-positive rate (sensitivity) and the false-positive rate, was calculated.^20^ All analyses were done using STATA (Version 15).

### Ethical Approval

We obtained ethical clearance from the Ministry of Health and Population, Nepal Health Research Council, Kathmandu, Nepal (NHRC #1620/2017). All respondents gave written informed consent before the interview and collection of anthropometric data. No treatment or incentive was given to the study participants.

## RESULTS

Selected background characteristics of the surveyed households and children aged 6–59 months are presented in Table 1. Children were on average aged 28 months; slightly less than half (43.7%) were aged 6–23 months, and the rest (56.2%) were aged 24–59 months. More than half (55.8%) of the children were male, and more than half (56.5%) resided in the hilly region of the country, versus the lowland plains and mountains of Nepal. Almost half (49.6%) of the surveyed children were from a socially excluded ethnic group. Among the children, 28.0% were stunted (low height-for-age). The overall prevalence of wasting based on WHZ was 10.5%, comprised of 2.1% SAM and 8.5% MAM, whereas only 0.4% SAM and 2.7% MAM were found based on MUAC.

**TABLE 1.** Background Characteristics of Children Aged 6–59 Months Surveyed on Undernutrition, Nepal

	No. (%), N=3,169
Sex	
Male	1,768 (55.8)
Female	1,401 (44.2)
Child age, months	
6–23	1,387 (43.7)
24–59	1,782 (56.2)
Ecological zone	
Mountain	389 (12.3)
Hill	1,790 (56.5)
Terai	990 (31.2)
Type of residence	
Urban	1,588 (50.1)
Rural	1,581 (49.9)
Caste/ethnicity	
Socially excluded	1,571 (49.6)
Brahmin/Chhetri	1,247 (39.3)
Others	351 (11.1)
Equity quintile	
Lowest	681 (21.5)
2nd lowest	906 (28.6)
Middle	745 (23.5)
2nd highest	638 (20.1)
Highest	199 (6.3)
Mother's education level	
Never attended school	693 (21.8)
Some primary school	439 (13.8)
Completed grade 5	243 (7.7)
Some secondary education	1,051 (33.2)
Completed secondary education	392 (12.4)
Completed class 12	289 (9.1)
Higher education	62 (1.9)
Prevalence of food insecurity	
Food secure	2,135 (67.4)
Mildly food insecure	497 (15.7)
Moderately food insecure	465 (14.7)
Severely food insecure	71 (2.2)
WHZ	
SAM (<−3 SD)	65 (2.1)
MAM <−2 SD to ≥−3 SD)	269 (8.5)
Normal (>−2 SD)	2,835 (89.4)
MUAC	
SAM (<115 mm)	12 (0.4)
MAM (≥115 mm to <125 mm)	87 (2.7)
Normal (>125 mm)	3070 (96.9)
Stunting	
Stunted	888 (28)
Not stunted (Normal)	2,279 (72)

Abbreviations: MAM, moderate acute malnutrition; MUAC, mid-upper arm circumference; SAM, severe acute malnutrition; SD, standard deviation; WHZ, weight-for-height/length z-score.

We found 13.6% sensitivity for SAM (MUAC <115 mm) and 21.0% sensitivity for MAM (MUAC ≥115 mm to <125 mm) with specificity of 99.7% and 91.2%, respectively. The total area of the ROC curve was 0.53 for the MUAC cutoff for SAM (<115 mm). The kappa value for the SAM cutoff was 9.0%. The total area under ROC curve was 0.56 for the MAM cutoff ≥115 mm to <125 mm. The kappa value for the MAM cutoff was 17.0%.

Comparison of ROC and kappa by child gender and age are shown in Table 2. The total AUC of the SAM cutoff for male and female children were 0.51 and 0.56, respectively. The kappa value for the SAM cutoff among boys was 4.0% and 16.0% for girls. The total AUC of the MAM cutoff for boys and girls was 0.52 and 0.62 with kappa values 11.0% and 24.0%, respectively.

**TABLE 2.** Area Under ROC Curve and Kappa Value to Compare MUAC and WHZ Cutoffs for Children Aged 6–59 Months, Nepal

	SAM (<115 mm)		MAM (≥115 to <125 mm)	
	Area Under ROC	Kappa, %	Area Under ROC	Kappa, %
Overall	0.53	9	0.56	17
Sex				
Male	0.51	4	0.52	11
Female	0.56	16	0.62	24
Child age, months				
6–23	0.61	12	0.61	25
24–59	0.51	6	0.53	7
Stunting				
Stunted	0.51	10	0.56	16
Not stunted	0.54	10	0.56	16

Abbreviations: MAM, moderate acute malnutrition; MUAC, mid-upper arm circumference; ROC, receiver operating characteristic curve; SAM, severe acute malnutrition; WHZ, weight-for-height/length z-score.

Among the children aged 6–23 months, 11.5% were found wasted including 8.9% with MAM and 2.6% with SAM using WHZ, but only 5.0% MAM and 0.7% SAM were identified as wasted using MUAC. Among children aged 24–59 months 9.8% were identified as wasted using WHZ including 8.2% with MAM and 1.6% with SAM, whereas using MUAC, only 0.7% and 0.2% of these children were diagnosed with MAM and SAM, respectively. The total AUC of the SAM cutoff was 0.61 and 0.51 for children aged 6–23 months and 24–59 months, respectively. The kappa value of the SAM cutoff for children aged 6–23 months was 12.0% and 6.0% for children aged 24–59 months. Likewise, the total AUC of the MAM cutoff for children aged 6–23 months and 24–59 months were 0.61 and 0.53, respectively. The kappa value of the MAM cutoff for children aged 6–23 months was 25.1% and 7.0% for children aged 24–59 months.

Among the children aged 6–23 months, 8.9% with MAM and 2.6% with SAM were identified as wasted using WHZ, but only 5.0% with MAM and 0.7% with SAM were identified as wasted using MUAC.

Table 3 shows the sensitivity, specificity, and Youden index at various MUAC cutoff values for diagnosing SAM. The optimum cutoff of MUAC for SAM was found to be 125 mm with a maximum Youden index of 49.9%. Similarly, the best cutoff point of MUAC for optimum diagnosis of MAM was found to be 132 mm with a Youden index 30.5% (Table 4).

**TABLE 3.** Sensitivity, Specificity, and Youden Index at Various Cutoffs of MUAC for SAM in Children Aged 6–59 Months, Nepal

MUAC, mm	Sensitivity, %	Specificity, %	Youden Index, %
105	4.0	100	4.0
106	4.1	100	4.1
107	4.8	100	4.8
108	5.1	100	5.1
109	5.4	100	5.4
110	6.9	100	6.9
111	7.6	100	7.6
112	9.1	100	9.1
113	11.2	100	11.2
114	12.8	99.8	12.6
115	13.6	99.7	13.3
116	14.3	99.0	13.3
117	18.9	99.1	18.0
118	21.6	98.7	20.3
119	24.3	98.5	22.8
120	28.9	98.3	27.2
121	34.5	98.1	32.6
122	35.9	98.0	33.9
123	43.6	96.6	40.2
124	46.8	96.4	43.2
125	53.7	96.2	49.9
126	59.0	87.2	46.2
127	66.0	73.0	39.0
128	69.0	56.3	35.3
129	72.0	46.1	31.8
130	90.1	41.0	31.1
131	93.0	37.5	30.5
132	93.5	33.3	26.8
133	93.1	31.5	24.6
134	93.2	28.5	21.7
135	97.6	26.2	23.8

Abbreviations: MUAC, mid-upper arm circumference; SAM, severe acute malnutrition.

**TABLE 4.** Sensitivity, Specificity, and Youden Index at Various Cutoffs of MUAC for MAM in Children Aged 6–59 Months, Nepal

MUAC, mm	Sensitivity, %	Specificity, %	Youden Index, %
115	0.2	100	0.2
116	0.6	100	0.6
117	1.2	100	1.2
118	3.4	100	3.4
119	3.9	99.5	3.4
120	5.8	98.0	3.8
121	9.4	97.1	6.5
122	12.0	96.4	8.4
123	14.3	95.2	9.5
124	17.0	94.1	11.1
125	21.0	91.2	12.2
126	33.2	87.3	20.5
127	39.2	84.3	23.5
128	45.2	80.4	25.6
129	51.2	76.8	28.0
130	61.2	69.3	29.5
131	64.3	65.4	29.7
132	66.6	63.9	30.5
133	68.9	57.0	25.9
134	71.2	51.0	22.2
135	71.6	49.2	20.8
136	74.3	46.3	20.6
137	75.8	44.2	20.0
138	77.6	40.1	17.7
139	79.6	37.5	17.1
140	81.6	34.5	16.1

Abbreviations: MAM, moderate acute malnutrition; MUAC, mid-upper arm circumference.

## DISCUSSION

This study compares the sensitivity and specificity of MUAC versus WHZ to identify wasting in children aged 6–59 months in Nepal, to contribute to local and global debates on how to ensure the timely and accurate diagnosis of wasting, a prerequisite to identifying and treating millions of children globally suffering from undernutrition. This is the first-ever study comparing the performance of MUAC and WHZ in Nepal to identify wasted children and among the few studies globally to use population-based, rather than hospital/treatment center-based, data to explore variations in the MUAC and WHZ indicators. This study looks at discrepancies in classification overall and then separately by child age and child gender. The percentage of wasting, as measured by WHZ, was found to be almost the same as the national prevalence found in the recent demographic and health survey done in 2016 (10.5% vs. 9.8%).^6^ Among these wasted children, we found that 2.1% were SAM and 8.5% were MAM, but when using MUAC we only found 0.4% SAM and 2.7% MAM. When comparing the 2 methods for identification, and using WHZ as the reference standard, the total AUC of MUAC for both SAM and MAM, showed that in 53% and 56%, respectively. These are close to an AUC of 0.5, which is the same as complete randomness, and suggests that the current MUAC cutoffs are poor tools for detecting wasting.

With WHZ as the reference standard, we found MUAC only had 13.6% sensitivity for SAM (MUAC <115 mm) and 21% sensitivity for MAM (MUAC ≥115 mm to <125 mm), with a specificity of 99.7% and 91.2%, respectively. This is consistent with various studies that have reported a very wide range of sensitivity of MUAC, ranging from 17.5% to 43.5% and consistently higher specificity.^6^^,^^9^^–^^11^^,^^21^ A study by Grellety et al.^21^ in a therapeutic feeding center that included 2,205 South Sudanese children concluded that MUAC <115 mm would have failed to identify 33.0% of the children with SAM, while 98.0% were identified by WHZ <−3 SD alone and 100% by MUAC <130 mm. A systematic review and meta-analysis by Grellety and Golden^22^ concluded that the use of MUAC alone to identify SAM children, versus using both WHZ and MUAC, would exclude many children from treatment and thus, result in an additional 300,000 SAM related deaths annually. In Asia, the evidence around these challenges with using MUAC to identify and treat malnourished children is building. Fiorentino et al.^23^ also found in Cambodia that the sensitivity of MUAC ranged from 6.5% to 32.9% in children with acute malnutrition and from 0.0% to 18.2% in children with severe acute malnutrition. According to Talapalliwar and Garg^24^ the sensitivity and specificity of MUAC <115 mm was 13.6% and 99.3%, respectively and the current cutoff can only capture a small proportion of all children with SAM, in the context of tribal populations in India. Our findings also show that many Nepalese children could be excluded by using only MUAC to identify wasting.

Similarly, since MUAC >115 mm is being used in Nepal as a single criterion to discharge children with SAM from outpatient therapeutic centers, proper nutritional rehabilitation will be missed, and thousands of Nepalese children will be discharged as cured who could still be suffering from undernutrition or at increased risk of relapse. Finally, many studies to date focus on SAM, but MAM management programs, especially blanket and targeted supplementary feeding programs in Nepal, use MUAC only. These findings show that a large number of children with MAM are being missed and not provided with nutrition rehabilitation, counseling, and support because MUAC was the only anthropometric measurement used. Existing literature suggests that MUAC-only based programs tend to identify significantly more girls and younger children than those identified by WHZ.^23^ In this study, the total AUC for male children was slightly lower than for female children (0.51 vs. 0.56), with kappa 4.0% and 16.0%, respectively, which indicates that MUAC captured faintly more wasted female children than male children. The ROC and kappa results in this study also suggest that MUAC may be a more useful approach for the diagnosis of both SAM and MAM in children aged less than 2 years than for older children. Prior studies also provide similar evidence of age variation in the ability of MUAC to detect both SAM and MAM.^23^^,^^25^^–^^27^ The age dependency may be due to variation of muscle mass with increasing age of children.

These findings show that a large number of children with MAM are being missed and not provided with nutrition rehabilitation, counseling, and support because MUAC was the only anthropometric measurement used.

In line with previous studies,^25^^,^^26^^,^^28^ the results of our Youden index analyses, which identify an ideal performance point by calculating the optimum level of sensitivity and specificity, suggest that current MUAC cutoffs must be increased to capture more cases of wasting. The optimum MUAC cutoff point to identify children with SAM was found to be 125 mm. Likewise, the best cutoff for MUAC to optimally identify children with MAM was found to be 132 mm. Talapalliwar and Garg^24^ also found the optimal cutoff of MUAC to be 125 mm for proper detection of SAM and 136 mm for diagnosing MAM. Laillou et al.^5^ suggested that a MUAC cutoff of 133 mm would allow the inclusion of more than 65% of children with a WHZ <−3 to be considered wasted. Similarly, a recent study by Tessema et al.^29^ in Ethiopia concluded that implementation of a MUAC-only screening program, using a cutoff of <115 mm for the identification of SAM, is unethical as it may lead to many children remaining undiagnosed and untreated. They suggested a MUAC cutoff <133 mm for optimum identification of children with SAM. Furthermore, consistent with our findings, Fiorentino et al.^23^ concluded that MUAC cutoffs by age group and gender should be revised for community-level screening and treatment of wasting.

### Limitations

A limitation of this study is that we could not analyze relationships between the screening tools and mortality, screening tools and response to treatment such as weight gain, or time for nutrition rehabilitation/graduation from treatment. We have used a cross-sectional dataset, which could not capture information on response to treatment or recovery/survival. Furthermore, screening and exclusion of children with edema were not done in these analyses, which could offer yet another interesting perspective on the prevalence of acute malnutrition. We used WHZ as a reference standard, but WHZ itself is not without its challenges for diagnosis and treatment of wasting, including the need for all health workers to be trained and standardized to ensure accuracy of measurement and for anthropometric equipment to be maintained in good condition.

## CONCLUSION

This study confirms that MUAC, using WHZ as the reference standard, can detect wasting but in only a small fraction of all wasted children, leaving the majority of wasting in Nepal undetected and untreated. The poor performance in terms of sensitivity and specificity confirms the need to either increase the MUAC cutoff values or adopt both MUAC and WHZ at every health facility and in acute malnutrition management programs for early, rapid, and accurate diagnosis and treatment of wasting in Nepal. Therefore, WHZ should also be used as the admission and discharge criteria, rather than MUAC alone, as a standalone anthropometric criterion at therapeutic centers. Additionally, the Government of Nepal and development partners will need to continue to work with communities to ensure that more children are brought to the health facilities where these criteria would be used. Furthermore, while evidence-based updating is done to policies and programs for the treatment of undernutrition, governments and development partners should continue to invest in prevention efforts. Multisectoral policies and programs focused on the prevention of all forms of child malnutrition should continue so that the prevalence of wasting does not remain above 10% for another 20 years. The findings of this study can help the Government of Nepal and development partners to update screening and management approaches for acute malnutrition to enable timely and proper nutrition rehabilitation of children aged under 5 years.

## Notes

**Peer Reviewed**

**First published online:** December 9, 2021.
